# Current scenario of organ donation and transplantation in Kerala, India

**DOI:** 10.3126/nje.v9i2.24679

**Published:** 2019-06-30

**Authors:** Ahammed Mekkodathil, Mohammad Asim, Brijesh Sathian, Elayedath Rajesh, Rajeev N Kumar, Padam Simkhada, Edwin van Teijlingen

**Affiliations:** 1 Injury prevention coordinator, Clinical Research, Trauma and Vascular Surgery, Surgery Department, Hamad General Hospital, Doha, Qatar.; 2 Academic Research Associate, Clinical Research, Trauma and Vascular Surgery, Surgery Department, Hamad General Hospital, Doha, Qatar.; 3 Assistant Professor, Schoool of Behavioural Sciences, Mahatma Gandhi University, Kerala, India; 4 Director & Associate Professor, Schoool of Behavioural Sciences, Mahatma Gandhi University, Kerala, India; 5 Associate Dean for the Faculty of Education Health and Community, Professor of International Public Health, Liverpool John Moores University, UK; 6 Professor, Centre for Midwifery, Maternal and Perinatal Health, Bournemouth University, Bournemouth, UK.

Organ transplantation has been an accepted mean of treating patients with severe organ failure in India for nearly five decades. However, the organ donation rate of people who have died in India is very low (0.26 per million population), and this low rate partly contributes to the deaths of 500,000 people every year due to lack of available organs [1]. Kerala, one of the southern states in India with 35 million inhabitants, claims some of the best health statistics in the country, in fact some are similar to those in the high-income countries, has a deceased organ donation rate of 1.03 per million population [2]. Although this rate is four times higher than the national rate, it remains lower than that of the neighboring state of Tamil Nadu which has the highest deceased organ donation rate in the country (1.9 per million population) [2]. Alarmingly, the deceased donor transplant data by Kerala Network for Organ Sharing (KNOS) revealed a significant decline in number of donations in recent years. In 2015, there were 218 major organ donations by 72 deceased donors whereas only 29 major organs were donated by 8 deceased donors in 2018, which suggests major limitations of the existing deceased organ donation programme at governmental level [3].

Lack of or negligible brain death declaration in many hospitals across the state was identified as one of the major reasons for delays or lack of organ donations. Working closely with Donation and Transplantation Institute of Spain, the Government of Kerala recently provided a training programme on Transplant Procurement Management for professionals (mainly neurologists and anesthetists) from all over the state [4]. The training programme included establishing brain death and best ways of obtaining consent from relatives for organ harvesting and transplantation [4]. Moreover, the government is currently planning to introduce a new position of Transplant Coordinator in every hospital in the state to facilitate deceased organ donations. Brain death declaration in Kerala is being performed by a board of medical experts which include two doctors from outside the hospital where the brain death patient was treated, and one should be a doctor employed in the government facility. In addition, now there is a regulation to videotape the apnea tests conducted for the assessment and diagnosis of brain death. These medical governance measures will ensure transparency in the process which will ultimately address the misconceptions about organ donation in the healthcare system that may exist among the population.

In addition to these efforts, another important area of focus for improving organ donation rates is addressing religious and superstitious beliefs about organ donation. Notably, no religious law prohibits their followers from donating their organs and tissues and so they are generally positive about organ donation. In 2012, The MOHAN Foundation organized a “one of its kind” multi-religious confluence titled “Sant Sangama” where religious leaders unanimously endorsed organ donation as the most supreme form of gift [5]. However, lack of education and awareness about organ donation, especially about religious edicts, were identified as important negative predictors in many settings. Therefore, the state authorities should support research projects exploring major factors influencing organ donation among general population and provide community-based interventions. It should also seek the possibilities of bringing nonprofit organizations, community-based organizations, and faith-based organizations in Kerala to a common platform to promote organ donation. The experts who participated in the International Conference on Mixed Method Research (ICMMR 2019) held at Mahatma Gandhi University Kerala, India, discussed the role of community-based participatory research approach in addressing such issues. Better research on beliefs and superstition in the community utilizing appropriate design will help to formulate better policies and population-wide health education programmes.
